# The Self-Expanding Symetis Acurate Does Not Increase Cerebral Microembolic Load When Compared to the Balloon-Expandable Edwards Sapien Prosthesis: A Transcranial Doppler Study in Patients Undergoing Transapical Aortic Valve Implantation

**DOI:** 10.1371/journal.pone.0108191

**Published:** 2014-10-07

**Authors:** Gabor Erdoes, Christoph Huber, Reto Basciani, Stefan Stortecky, Stephan Windecker, Peter Wenaweser, Thierry Carrel, Balthasar Eberle

**Affiliations:** 1 Department of Anesthesiology and Pain Therapy, Inselspital, University Hospital Bern, Bern, Switzerland; 2 Department of Cardiovascular Surgery, Inselspital, University Hospital Bern, Bern, Switzerland; 3 Department of Cardiology, Inselspital, University Hospital Bern, Bern, Switzerland; Colorado State University, United States of America

## Abstract

**Objectives:**

The aim of this study was to quantify potential differences in count, frequency and pattern of high-intensity transient signals (HITS) during transapical transcatheter aortic valve implantation (TA-TAVI), by comparing the Symetis Acurate TA (SA) with the balloon-expandable Edwards Sapien XT (ES) system.

**Background:**

Recently, the Symetis Acurate TA revalving system has been introduced for TA-TAVI. The Symetis Acurate TA aortic bioprosthesis is self-expanding and is deployed by a specific two-step implantation technique. Whether this novel method increases the load of intraprocedural emboli, detected by transcranial Doppler ultrasound (TCD) as HITS, or not is not clear.

**Methods:**

Twenty-two patients (n = 11 in each study arm, median logistic EuroScore 20%, median STS score 7%) displayed continuous TCD signals of good quality throughout the entire TA-TAVI procedure and were included in the final analysis. Data are presented as median with interquartile ranges.

**Results:**

No significant differences were detected in total procedural or interval-related HITS load (SA: 303 [200; 594], ES: 499 [285; 941]; p = 0.16). With both devices, HITS peaked during prosthesis deployment (PD), whereas significantly fewer HITS occurred during instrumentation (SA: p = 0.002; ES: <0.001) or post-implantation PI (SA: p = 0.007; ES: <0.001). PD-associated HITS amounted to almost half of the total HITS load. One patient suffered new disabling stroke at 30 days. Thirty-day mortality amounted to 13.6% (3 of 22 patients).

**Conclusions:**

Simplified transapical delivery using the self-expanding SA device does not increase HITS, despite of a two-step deployment technique with more interactions with the native aortic valve, when compared to the balloon-expandable ES valve. The similarity in HITS count, frequency and pattern with the two systems suggests a common mechanism for the release of cerebral microemboli.

## Introduction

In selected high-risk patients with severe aortic valve stenosis, early and late results of the PARTNER trial allow for a direct comparison of event rates of stroke and transient ischemic attack between transcatheter aortic valve implantation (TAVI) and surgical aortic valve replacement (SAVR) [1]–[3]. Results indicate that the incidence of stroke after TAVI is characterized by an early and a late (>2 years) risk period. TAVI appears to be associated with a numerical increase of early cerebrovascular events at 30 days of follow-up, when compared with SAVR (major stroke 3.8% vs. 2.1%, p = 0.20) which is still available at one year of follow-up (5.1% vs. 2.4%, p = 0.07) [2]. In contrast, the risk for late stroke does not appear to be related to the technique of valve replacement (stroke at 2 years: TAVI 7.7% vs. SAVR 4.9%; p = 0.17) [3].

The passage of stiff guidewires, catheters and the delivery system through the aortic arch (when TAVI is performed via a transfemoral approach) and through the degenerative aortic valve as well as, but also balloon aortic valvuloplasty and prosthesis deployment may detach atheromatous or calcified debris, and may cause cerebral microembolization with occasionally devastating complications [4]–[6]. This etiology of intraprocedural stroke is supported by diffusion-weighted magnet resonance imaging (DW-MRI) and transcranial Doppler ultrasound (TCD) studies. Following TAVI, new but clinically silent foci of restricted diffusion are regularly detected at cerebral DW-MRI [7]–[10]. Continuous periprocedural TCD monitoring has identified instrumentation and prosthesis deployment as critical procedural steps in TAVI, with peaks of high-intensity transient signals (HITS) attributable to showers of particulate or gaseous microemboli passing the middle cerebral artery (MCA) en route to the brain [11]–[13].

Rapid innovation of self-expanding TAVI devices [14] and implantation techniques may affect device-specific intra-procedural cerebral microembolic load. However, little or no information is available about such effects, especially in comparison to established TAVI systems.

As one of these innovations, the self-expanding Symetis ACURATE TA system has been designed for the transapical access and has received CE mark approval at the end of 2011. The device combines various new prosthesis- and implantation-related features designed to facilitate precise and reproducible deployment. The prosthesis is delivered by a two-step deployment technique, allowing tactile feedback control by interaction of the Acurate frame with the native aortic valve. This technique increases deployment accuracy, but might also increase friction and hence, risk of cerebral thromboembolism.

The aim of the current study was to investigate the incidence, rate and temporal pattern of HITS during transapical TAVI (TA-TAVI), using either the new Symetis ACURATE (SA) device or the conventional Edwards SAPIEN (ES) prosthesis and implantation technique.

## Methods

### STUDY DESIGN

Consecutive patients undergoing TA-TAVI have been analyzed for the purpose of this study. The patients are a subset of our institutional prospective single-center registry initiated in July 2007. Selection of the SA or ES device was non-randomized and guided by patient and device parameters. All patients had given written informed consent to undergo TA-TAVI under general anesthesia with invasive and noninvasive monitoring, and for the scientific use of their anonymized data. Approval for retrospective study was given by the Cantonal Ethics Committee Bern, and granted by the Institutional Research Directorate. The corresponding author takes full responsibility for the integrity of the data. All authors have read the manuscript and agree with the present version.

### PATIENTS

During the period from March 2012 to February 2013, thirty-one patients underwent TA-TAVI at our institution. Sixteen patients were treated with the SA, and 15 patients with the ES system. Patients were considered for a TA-TAVI procedure based on clinical, echocardiography and computed tomography findings by the institutional heart team consisting of interventional cardiologists and cardiac surgeons. Selection criteria for TA-TAVI were severe symptomatic aortic stenosis (calculated aortic valve area <1 cm^2^; aortic valve mean pressure gradient >40 mmHg on echocardiography), age >80 years, and/or a logistic EuroScore ≥15%, or age >70 years with appreciated high or prohibitive risk of morbidity or/and mortality for SAVR. Selection of the SA or ES device occurred at the clinical discretion of the institutional heart team based on aortic root anatomy, distribution of valvular calcification and technical characteristics of the prostheses.

### TRANSAPICAL AORTIC VALVE IMPLANTATION PROCEDURE

Procedural characteristics and the design of the implanted devices have been described elsewhere [15], [16]. All procedures were performed in a standardized fashion as reported in detail by others and our group [16], [17].

Depending on distinct anatomical characteristics, either a Symetis (Symetis ACURATE TA, Symetis Inc., Ecublens, Switzerland) or an Edwards (Edwards SAPIEN XT, Edwards Lifesciences Inc., Irvine, CA, USA) was selected. In all patients TA-TAVI included echocardiography-guided anterolateral mini-thoracotomy, access site preparation, insertion of the apical introducer and balloon aortic valvuloplasty of the calcified native aortic valve during rapid ventricular pacing (RVP). This was followed by angiography-guided delivery of the TA-TAVI device. The balloon-expandable ES was deployed by a single implantation step during RVP. The self-expanding SA device was deployed by a specific two-step implantation technique. The first step consisted of partial release with unsheathing of the stabilization arches and the upper crown, to obtain initial valve positioning with optimal axial alignment. After commissural alignment under gentle retrograde pull, the upper crown was reversibly brought in touch with the calcified leaflets of the native aortic valve until tactile feedback could be felt. In a second step, final placement was achieved with full prosthesis deployment during stable tactile feedback. RVP could be initiated at the surgeon’s preference but was not mandatory.

All TA-TAVI procedures were performed in general endotracheal anesthesia using propofol and remi-fentanil. Continuous anesthesia monitoring consisted of ECG, pulse oximetry, capnography, invasive arterial and central venous pressure, transesophageal echocardiography, processed EEG (BIS) and bilateral transcranial Doppler sonography. Postoperatively, patients were either extubated in the angiography suite, or remained intubated until circulatory and respiratory stabilization. Thereafter, all patients were transferred to the intensive care unit.

Intraprocedural anticoagulation consisted of IV bolus administration of heparin (70–100 IU/kg) with the goal to achieve an activating clotting time of >250 s throughout the implantation procedure. The oral antiplatelet regimen was started with acetylsalicylic acid (100 mg/d) and a loading dose of 300 mg clopidogrel, which was administered six hours after apical access closure. Patients were discharged with a prescription of acetylsalicylic acid 100 mg/day indefinitely and clopidogrel 75 mg/day for 3–6 months. If oral anticoagulation was indicated, warfarin was combined with either acetylsalicylic acid or clopidogrel.

### TRANSCRANIAL DOPPLER ULTRASONOGRAPHY AND EMBOLUS DETECTION

Transcranial Doppler ultrasonography and HITS detection were performed as described previously by our group [11]. Two-MHz pulsed-wave transducers (Spencer Technologies, Seattle, WA, USA) were placed bilaterally at the posterior temporal bone windows, using a probe-holding headframe system for continuous signal acquisition (Marc 600 series; Spencer Technologies, Seattle, WA, USA). Power output and gain settings were adjusted on the TCD machine (ST^3^, Model# PMD 150; Spencer Technologies, Seattle, WA, USA) to provide an optimal signal-to-noise ratio. Sample volume was set to 3 mm for all recordings. Using the 33 gate Power M-mode function for rapid vessel localization, characteristic blood flow velocity spectra were detected with high signal quality by insonation of the MCA at a depth between 46 and 58 mm. Automated HITS monitoring used the multi-depth embolic detection mode with artefact rejection. A post-procedural analysis of all TCD recordings was conducted by one of the authors (*GE*) in order to confirm the quantity and quality of microembolic signals.

As the primary endpoint of the analysis, the cumulative count of HITS and the specific HITS load related to the following predefined procedural intervals were analyzed:

1. Instrumentation (i.e., placement of catheters and guidewires) prior to and until balloon aortic valvuloplasty of the native aortic valve, (IN)
2. Balloon aortic valvuloplasty of the native aortic valve, (BAV)
3. Prosthesis deployment (PD) and
4. Post-implantation period including any post-dilatation maneuvers (PI).

The duration of each period was documented and also validated using the interventional protocols. Phase-specific HITS rate was defined as the ratio of HITS count per specific interval duration (count/min per interval).

### NEUROLOGIC ASSESSMENT

All patients were examined (board-certified anesthesiologist, *GE*) prior to the procedure and after TA-TAVI between post-procedural days 1 and 6 by performing a gross neurological status and by confusion assessment using the CAM-ICU questionnaire [18]. Any abnormal findings in the neurological assessment triggered consultation of a specialist neurologist and if appropriate, diagnostic neuroimaging.

### STATISTICAL ANALYSIS

Results are presented as number, percentage or median with quartiles [q25^th^; q75^th^].

Demographic data were compared using contingency table analysis for categorical variables, and analysis of variance (ANOVA) for continuous measures. HITS counts were not normally distributed, and were thus compared between different procedural steps using Friedman Repeated Measures ANOVA on ranks; for comparison between the two prosthesis types, Mann-Whitney rank sum test was used. p<0.05 was considered significant. Analyses were performed using SigmaPlot for Windows, Version 10.0 and SigmaStat for Windows, Version 3.0 (Systat Software, Inc., Germany).

## Results

### PATIENT POPULATION AND PROCEDURAL DATA

Of 31 patients undergoing TA-TAVI during the observation period, 22 patients (71%) displayed MCA spectra of good quality throughout the entire procedure, and were included in the final analysis. SA and ES implantations were equally distributed in each study arm (n = 11).

In the remaining 9 patients, TCD monitoring was not performed either due to logistic reasons (4/31) or due to absence of an appropriate temporal bone window (3/31), or data acquisition was terminated prematurely after loss of the MCA signal during the procedure (2/31).

Baseline characteristics and procedural data of the study population are summarized in tables 1. and 2. Except from an older age (p = 0.04) and a higher prevalence of coronary artery disease (p = 0.01) in patients undergoing ES implantation, baseline data did not show differences between groups SA and ES.

**Table 1:** Patient characteristics.

	Overalln = 22	Symetis ACURATE TAn = 11	Edwards SAPIEN XTn = 11	p
Age, yr.	84(80;85)	83(76;84)	84(82;86)	0.04*
Female sex	10(45)	7(63)	3(27)	0.10
BMI, kg/m^2^	25(23;29)	26(24;29)	24(20;29)	0.29
NYHA class	3(3;3)	3(3;3)	3(3;3)	0.20
Logistic EuroSCORE, %	20(13;37)	28(17;41)	19(11;26)	0.23
STS Score, %	7(6;10)	7(6;10)	8(7;10)	0.86
Arterial hypertension	18(81)	9(81)	9(81)	1.00
Hyperlipidemia	15(68)	7(63)	8(73)	0.68
Diabetes mellitus	5(23)	2(18)	3(27)	0.65
Smoking	10(45)	7(63)	3(27)	0.10
Atrial fibrillation	7(32)	3(27)	4(36)	0.68
Coronary artery disease	17(77)	6(54)	11(100)	0.01*
Ejection fraction	52(35;63)	55(37;64)	45(36;62)	0.54
Carotid stenosis >50%	5(19)	3(27)	2(18)	0.65
History of stroke	4(15)	1(10)	3(27)	0.30
Preoperative GCS	15(15;15)	15(15;15)	15(15;15)	1.00
Aortic valve area, cm^2^	0.5(0.5;0.8)	0.6(0.4;0.8)	0.5(0.5;0.7)	0.83
Mean transaortic gradient, mmHG	40(24;50)	32(24;50)	45(24;52)	0.39

Abbreviations: BMI, body mass index; NYHA class, New York Health Association class of the severity of heart failure; STS score, Society of Thoracic Surgeons risk score of perioperative mortality and morbidity; GCS, Glasgow Coma Scale.

Values are presented as median (Q1; Q3) and n (%).

*significant difference (Mann-Whitney Rank Sum Test).

In the SA group, the majority of the patients received size M prostheses (73%). In group ES, sizes 26 mm and 29 mm were implanted in 90% of the cases. Immediate implantation and device success was 100%. Delirium occurred in 7/22 (32%) patients, and disabling stroke in 1/22 (4.5%). The affected patient suffered an ischemic stroke on the day of TAVI as a consequence of severe carotid artery stenosis). Hemispheric HITS count and total HITS occurrence in this patient, however, were not different from the cohort’s (total HITS count: MCA left/right 103/97). All-cause 30-day mortality was 3/22 (13.6%). Intra- and post-procedural data as well as adverse outcomes were not significantly different between SA and ES groups (table 2).

**Table 2:** Procedural data.

	Overall	Symetis ACURATE TA	Edwards SAPIEN XT	p
Procedure time, min				
IN	29(27;35)	28(25;37)	32(28;35)	0.86
BAV	1(1;2)	1(1;2)	1(1;2)	0.67
PD	3(3;4)	3(2;3)	3(3;3)	0.94
PI	4(2;7)	5(2;11)	4(2;5)	0.41
Total	39(36;50)	40(35;49)	39(37;50)	0.95
Prosthesis size, mm				
Symetis S/Edwards 23		2(18)	1(10)	
Symetis M/Edwards 26		8(73)	5(45)	
Symetis L/Edwards 29		1(10)	5(45)	
Acute implantation success	22(100)	11(100)	11(100)	1.00
Re-ballooning	4(18)	3(27)	1(10)	0.30
Coronary intervention				
RIVA	2(10)	1(10)	1(10)	1.00
RCA	1(5)	0(0)	1(10)	0.35
Administered heparin, kIU	5(5;5)	5(5;5)	5(5;6)	0.94
Radiocontrast dye volume, ml	154(113;158)	119(95;168)	155(112;184)	0.51
Fluoroscopy time, min	9(7;10)	9(8;12)	8(7;10)	0.26
General anesthesia, n (%)	22(100)	11(100)	11(100)	1.00
Access-related complications	0(0)	0(0)	0(0)	1.00
Hemodynamic instability	2(10)	1(10)	1(10)	1.00
Extubation in angio suite	8(36)	7(64)	1(10)	0.25
ICU stay, hrs.	21(17;23)	20(14;46)	21(15;22)	0.31
Delirium n (%)	7(32)	3(27)	4(36)	0.68
Stroke at 30-days n (%)	1(5)	0(0)	1(10)	0.35
30-day mortality n (%)	3(14)	1(10)	2(18)	0.57

Abbreviations: IN, instrumentation; BAV, balloon aortic valvuloplasty; PD, prosthesis deployment; PI, post-implantation period; S, small size;

M, medium size; L, large size; RIVA, ramus interventricularis anterior; RCA, right coronary artery; ICU, intensive care unit.

Values are presented as median (Q1; Q3) and n (%).

### INTRAPROCEDURAL HITS: EFFECTS OF DEVICE AND PHASE ON LOAD AND RATE

**Figure 1:**
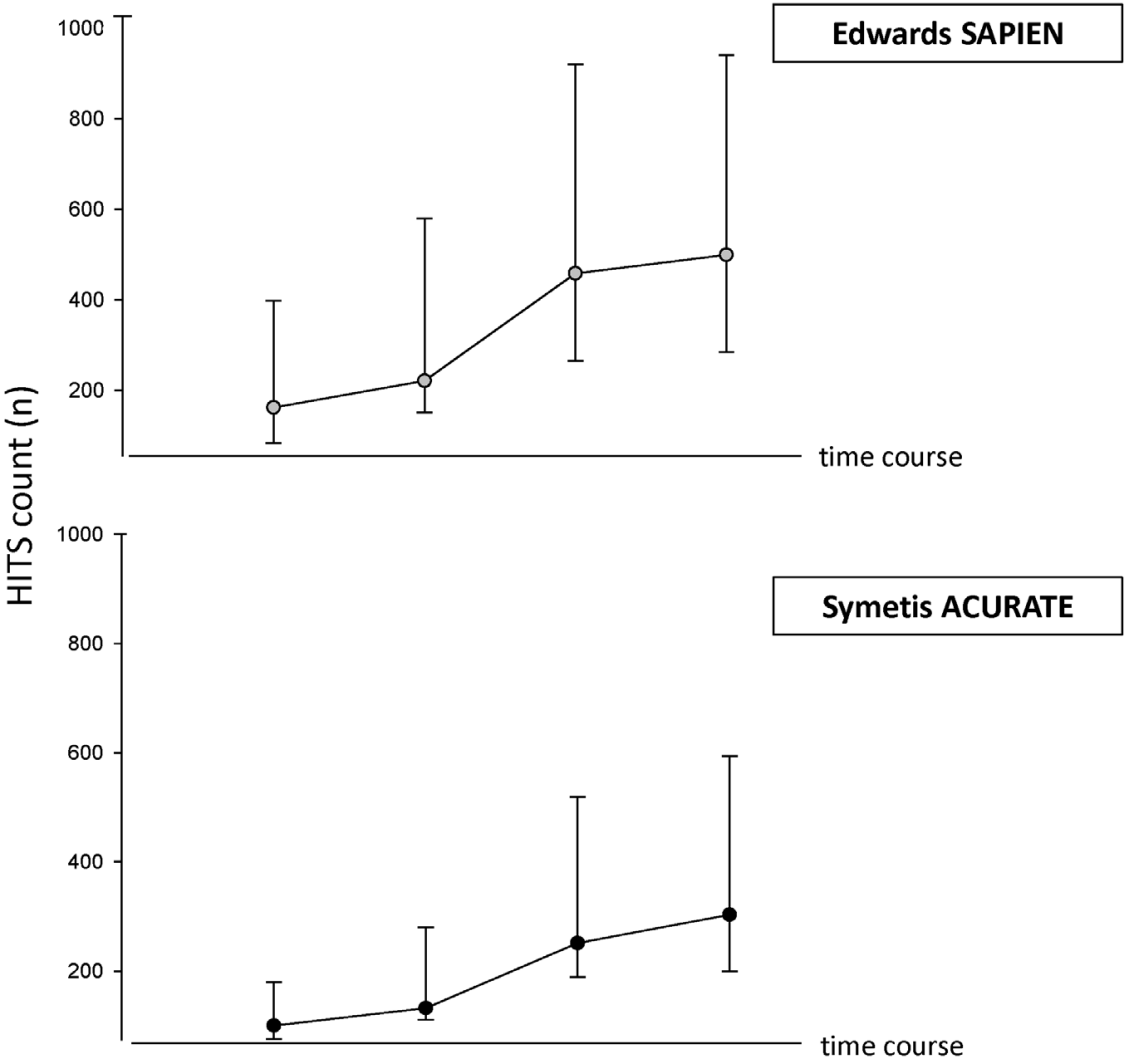
Cumulative HITS load during TA-TAVI procedure according to type of prosthesis. Abbreviations: HITS, high intensity transient signal. Data are presented as median with interquartile ranges.

**Figure 2:**
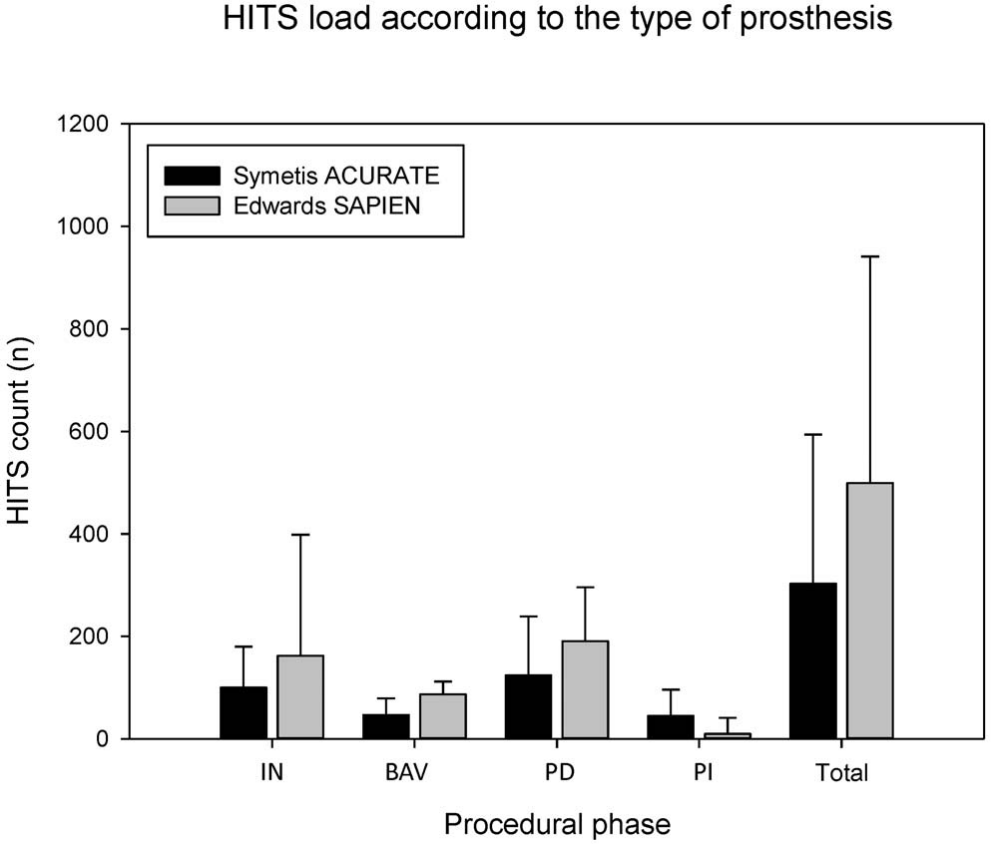
Interval-specific HITS load according to type of prosthesis. Abbreviations: HITS, high intensity transient signal; IN, instrumentation; BAV, balloon aortic valvuloplasty; PD prosthesis deployment; PI, post-implantation period. Data are presented as median with 75% interquartile range.

Total intraprocedural cumulative HITS count did not significantly differ between devices (SA 303 [200; 594]; ES 499 [285; 941], p = 0.16), nor differed the interval-specific HITS load between SA und ES (figure 1 and figure 2). This resulted in a similar distribution pattern of HITS for the two procedures. In both groups HITS peaked during deployment, amounting to almost half of the total HITS count, with instrumentation following second in terms of HITS count. The smallest HITS load was observed during post-implantation period (figure 2).

Total as well as interval-specific HITS load showed symmetrical distribution to left and right brain hemisphere, and hence, similar time patterns. The devices did not significantly differ from each other when interval-specific HITS load was related to interval duration (HITS per min of interval, HITS rate) (table 3).

**Table 3:** HITS per interval minute according to the type of TAVI prosthesis.

Procedural phase	Symetis ACURATE TA	Edwards SAPIEN XT	P
IN	3(2;6)	6(3;9)	0.13
BAV	44(24;56)	47(19;112)	0.83
PD	41(21;80)	64(33;148)	0.43
PI	5(1;14)	3(0;10)	0.41
Total	6(5;19)	13(9;18)	0.21

Abbreviations: IN, instrumentation; BAV, balloon aortic valvuloplasty; PD, prosthesis deployment; PI, post-implantation.

Values are presented as median (Q1; Q3).

**Figure 3:**
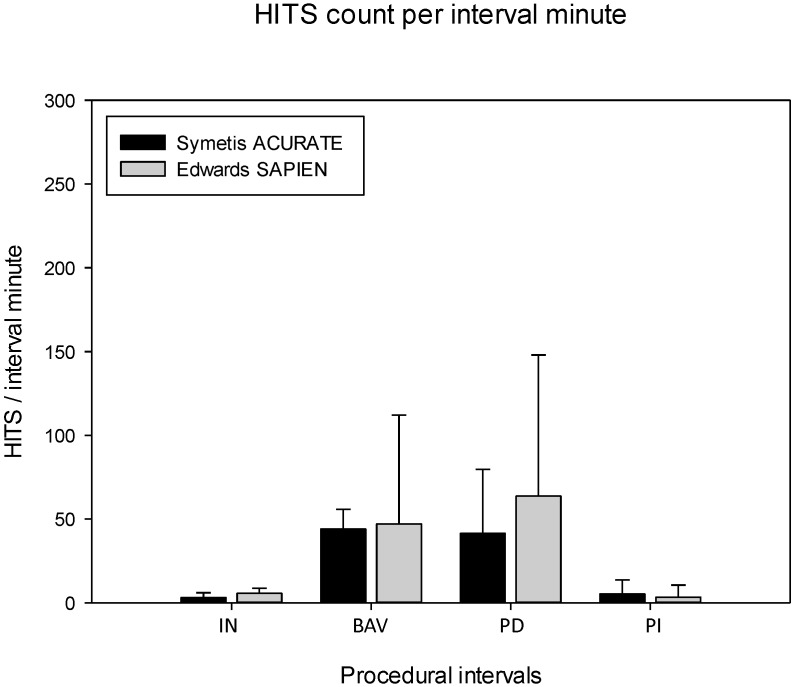
HITS load per interval minute according to type of prosthesis. Abbreviations: HITS, high intensity transient signal; IN, instrumentation; BAV, balloon aortic valvuloplasty; PD prosthesis deployment; PI, post-implantation period. Data are presented as median with 75% interquartile range.

In contrast, the procedural phase itself had a much larger impact: comparisons between procedural intervals demonstrated clearly that valvuloplasty (BAV) and deployment (PD) both had high and very similar phase-specific HITS rates, whereas instrumentation (IN) and post-implantation (PI) had a comparably low HITS rate in common (table 3, 4 and figure 3).

**Table 4:** Impact of procedural interval on HITS per interval minute according to the type of TAVI prosthesis.

Procedural phase	Symetis ACURATE TA	Edwards SAPIEN XT
IN vs. BAV	0.002*	<0.001*
IN vs. PD	<0.001*	<0.001*
IN vs. PI	0.694	0.470
BAV vs. PD	0.647	0.393
BAV vs. PI	0.045*	<0.001*
PD vs. PI	0.007*	<0.001*

Abbreviations: IN, instrumentation; BAV, balloon aortic valvuloplasty; PD, prosthesis deployment; PI, post-implantation.

*Significant difference in the values (Mann-Whitney Rank Sum Test).

## Discussion

This study investigated and compared count, frequency and intraprocedural pattern of HITS during TA-TAVI with either the self-expanding Symetis ACURATE TA revalving system or the balloon-expandable Edwards Lifesciences SAPIEN XT setup.

The main findings are, first, that both transapical devices release a similar total and interval-specific HITS load during and immediately after implantation. Second, deployment of the prosthesis is by far the period during which most HITS are generated. Independent of the device, balloon aortic valvuloplasty and valve deployment are associated with high and very similar HITS rates, although their duration and absolute HITS count differ.

We observed no difference in procedural and clinical outcomes (e.g., acute device success, access-related complications, neurologic morbidity, mortality) between SA and ES, although these were not endpoints in our small series.

Reduction of the microembolic load generated by TA-TAVI is an essential contribution to improving patient outcomes. New TA-TAVI devices may offer better deployment accuracy or ease of handling, but very little is known about the impact of these changes on cerebral microembolic release. The SA system features single-operator tactile feedback-guided deployment, but by this, may intensify device-calcification interaction prior to final implantation [15], [19]. This led us to hypothesize that this mode of implantation could result in a higher rate of cerebral microembolism when compared to the balloon-expandable single-shot release of the ES device.

In fact, no significant difference was found between the devices. SA produced a HITS rate and pattern very similar to that of the ES prosthesis. Several reasons may explain these results: user experience with the SA device is shorter than with ES technology, which is implanted already for 7 years in our institution. Thus, an operator-dependent increase in embolic load might still be produced by SA. A controlled prospective study with adequate power and at comparable points in the teamś learning curves may be necessary to observe true device-related differences. In addition, as we did not systematically perform non-contrast enhanced CT scan images, we are not able to investigate the association of overall aortic root calcification and its impact on HITS. However, at this point in time there is no clear evidence for an association between calcification amount or distribution and a higher rate of HITS during TA-TAVI, but will be definitely addressed in future studies. A further limitation of the study is related to the method of HITS detection. HITS were detected automatically using the automated multi-depth embolic detection software with artifact rejection. In this regard HITS detection has been performed according to the manufacturer’s specifications and current standards published in literature on this area. Nevertheless, our equipment was not able to differentiate between gaseous and particulate emboli. Therefore, the results do not allow to make statements on the quality of HITS. In this regard, it is possible that SA and ES exhibited different counts of particulate or gaseous HITS.

In view of our sample size we did not expect to see major differences because of the relatively low event rate of stroke or mortality. Although type II error due to low power (β = 0.278) is a clear limitation of the present series, our data appear useful for planning controlled comparative trials in the future. Second, the clinical outcome of patients treated with SA or ES was similar, therefore it is not surprising that the total HITS count is comparable. Apparently, neither the design improvements nor the seemingly advantageous two-step implantation of the SA device influence the mechanism of microemboli generation during TA-TAVI.

Today, there is still a very limited number of TCD studies, although the number of TA-TAVI procedures increases rapidly. None so far provided information about HITS occurrence with the SA device [7], [10]–[13], [20]. Reports on ES type prostheses agree on the fact that HITS occur regularly during TA-TAVI, and that an increased load of HITS is typically released during prosthesis deployment. Another device, the self-expanding CoreValve Revalving System (Medtronic Inc., Minneapolis, MN, USA), appears associated with even more marked HITS events during deployment and post-implantation [11]. Thus, in our opinion, the similarity in periprocedural HITS patterns between a variety of aortic prosthesis types, access routes and deployment techniques supports common pathomechanisms for most of the cerebral microembolic load during transcatheter aortic valve implantation.

Matching the present results to those of a previous study from our group allows to compare HITS patterns, owing to an identical definition of procedural phases [11]. Since this study, total procedural duration has decreased due to growing operator experience. Specifically, instrumentation and post-implantation periods became shorter. In contrast, valvuloplasty and deployment still take quite a similar absolute amount of time during recent procedures when compared to the past. They also release HITS at a similarly high rate. Such observations rather suggest *operator-independent, intervention-related HITS generation during TA-TAVI*. The procedural phase appears to be the leading determinant of the rate of microemboli release to the brain.

We conclude that balloon valvuloplasty and valve deployment are still the major contributors of intraprocedural HITS generation, most likely due to mechanical friction of the introducer system and/or the device itself within the aortic root. In this regard, the novel Symetis ACURATE TA system showed at least no inferiority to transapically implanted Edwards SAPIEN XT valves.

The results of this study indicate that the device-specific implantation technique and stent configuration of the Symetis ACURATE TA prosthesis are not associated with a substantial increase or decrease of cerebral microembolism. The similarity in HITS patterns of a variety of devices supports the hypothesis of a common pathomechanism for cerebral microembolism in TA-TAVI. Most probably this is related to hardware-tissue friction in the aortic root during balloon valvuloplasty and valve deployment. The evolution of next-generation TA-TAVI prostheses should also focus on improved deployment techniques which are less traumatic when performed in degenerated and calcified surfaces of the aortic root.

## References

[1] Leon MB, Smith CR, Mack M, Miller DC, Moses JW, et al (2010) Transcatheter aortic-valve implantation for aortic stenosis in patients who cannot undergo surgery. N Engl J Med 363: 1597–1607. 20961243 10.1056/NEJMoa1008232

[2] Smith CR, Leon MB, Mack MJ, Miller DC, Moses JW, et al (2011) Transcatheter versus surgical aortic-valve replacement in high-risk patients. N Engl J Med 364: 2187–98. 21639811 10.1056/NEJMoa1103510

[3] Kodali SK, Williams MR, Smith CR, Svensson LG, Webb JG, et al (2012) Two-year outcomes after transcatheter or surgical aortic-valve replacement. N Engl J Med 366: 1686–95. 22443479 10.1056/NEJMoa1200384

[4] Omran H, Schmidt H, Hackenbroch M, Illien S, Bernhardt P, et al (2003) Silent and apparent cerebral embolism after retrograde catheterisation of the aortic valve in valvular stenosis: a prospective, randomised study. Lancet 361: 1241–46. 12699950 10.1016/S0140-6736(03)12978-9

[5] Eggebrecht H, Schmermund A, Voigtländer T, Kahlert P, Erbel R, et al (2012) Risk of stroke after transcatheter aortic valve implantation (TAVI): a meta-analysis of 10,037 published patients. EuroIntervention 8: 129–38. 22391581 10.4244/EIJV8I1A20

[6] Stortecky S, Wenaweser P, Windecker S (2012) Transcatheter aortic valve implantation and cerebrovascular accidents. Eurointervention 8: Q60–69. 22995113 10.4244/EIJV8SQA11

[7] Rodes-Cabau J, Dumont E, Boone RH, Larose E, Bagur R, et al (2011) Cerebral embolism following transcatheter aortic valve implantation: comparison of transfemoral and transapical approaches. J Am Coll Cardiol 57: 18–28. 21185496 10.1016/j.jacc.2010.07.036

[8] Kahlert P, Knipp SC, Schlamann M, Thielmann M, Al-Rashid F, et al (2010) Silent and apparent cerebral ischemia after percutaneous transfemoral aortic valve implantation: a diffusion-weighted magnetic resonance imaging study. Circulation 121: 870–78. 20177005 10.1161/CIRCULATIONAHA.109.855866

[9] Ghanem A, Müller A, Nähle CP, Kocurek J, Werner N, et al (2010) Risk and fate of cerebral embolism after transfemoral aortic valve implantation: a prospective pilot study with diffusion- weighted magnetic resonance imaging. J Am Coll Cardiol 55: 1427–32. 20188503 10.1016/j.jacc.2009.12.026

[10] Arnold M, Schulz-Heise S, Achenbach S, Ott S, Dörfler A, et al (2010) Embolic cerebral insults after transapical aortic valve implantation detected by magnetic resonance imaging. J Am Coll Cardiol Interv 3: 1126–32. 10.1016/j.jcin.2010.09.008 21087747

[11] Erdoes G, Basciani R, Huber C, Stortecky S, Wenaweser P, et al (2012) Transcranial Doppler-detected cerebral embolic load during transcatheter aortic valve implantation. Eur J Cardiothorac Surg 41: 778–83. 22423058 10.1093/ejcts/ezr068

[12] Kahlert P, Al-Rashid F, Döttger P, Mori K, Plicht B, et al (2012) Cerebral embolization during transcatheter aortic valve implantation: a transcranial Doppler study. Circulation 126: 1245–55. 22899774 10.1161/CIRCULATIONAHA.112.092544

[13] Reinsfelt B, Westerlind A, Ioanes D, Zetterberg H, Fredén-Lindqvist J, et al (2012) Transcranial Doppler microembolic signals and serum marker evidence of brain injury during transcatheter aortic valve implantation. Acta Anaesthesiol Scand 56: 240–47. 22092012 10.1111/j.1399-6576.2011.02563.x

[14] Huber CH, Cohn LH, von Segesser LK (2005) Direct-access valve replacement a novel approach for off-pump valve implantation using valved stents. J Am Coll Cardiol 46: 366–70. 16022969 10.1016/j.jacc.2005.04.028

[15] Walther T, Dewey T, Borger M, Kempfert J, Linke A, et al (2009) Transapical aortic valve implantation: step by step. Ann Thorac Surg 87: 276–83. 19101311 10.1016/j.athoracsur.2008.08.017

[16] Kempfert J, Rastan AJ, Beyersdorf F, Schonburg M, Schuler G, et al (2011) Transapical aortic valve implantation using a new self-expandable bioprosthesis: initial outcomes. Eur J Cardiothorac Surg 40: 1114–19. 21924620 10.1016/j.ejcts.2011.01.078

[17] Buellesfeld L, Wenaweser P, Gerckens U, Mueller R, Sauren B, et al (2010) Transcatheter aortic valve implantation: predictors of procedural success – the Siegburg-Bern expericence. Eur Heart J 31: 984–91. 20038513 10.1093/eurheartj/ehp570

[18] Inouye SK, van Dyck CH, Alessi CA, Balkin S, Siegal AP, et al (1990) Clarifying confusion: the confusion assessment method. A new method for detection of delirium. Ann Intern Med 113: 941–8. 2240918 10.7326/0003-4819-113-12-941

[19] Kempfert J, Treedeb H, Rastan AJ, Schönburg M, Thielmann M, et al (2013) Transapical aortic valve implantation using a new self-expandable bioprosthesis (ACURATE TA™): 6-month outcomes. Eur J Cardiothorac Surg 43: 52–56. 22491663 10.1093/ejcts/ezs139

[20] Drews T, Pasic M, Buz S, Unbehaun A, Dreysse S, et al (2011) Transcranial Doppler sound detection of cerebral microembolism during transapical aortic valve implantation. Thorac Cardiovasc Surg 59: 237–42. 21442580 10.1055/s-0030-1250495

